# Cytokeratins Biosensing Using Tilted Fiber Gratings

**DOI:** 10.3390/bios8030074

**Published:** 2018-08-03

**Authors:** Médéric Loyez, Jacques Albert, Christophe Caucheteur, Ruddy Wattiez

**Affiliations:** 1 Proteomics and Microbiology Department, University of Mons, Champ de Mars 6, 7000 Mons, Belgium; ruddy.wattiez@umons.ac.be; 2 Department of Electronics, Carleton University, Mackenzie Building 5170, 1125 Colonel by Drive, Ottawa, ON K1S 5B6, Canada; jacquesalbert@cunet.carleton.ca; 3 Electromagnetism and Telecommunication Department, University of Mons, Bld Dolez 31, 7000 Mons, Belgium; christophe.caucheteur@umons.ac.be

**Keywords:** optical fiber, fiber Bragg grating, immunosensing, cytokeratin, cancer biomarker, diagnosis

## Abstract

Optical fiber gratings have widely proven their applicability in biosensing, especially when they are coupled with antibodies for specific antigen recognition. While this is customarily done with fibers coated by a thin metal film to benefit from plasmonic enhancement, in this paper, we propose to study their intrinsic properties, developing a label-free sensor for the detection of biomarkers in real-time without metal coatings for surface plasmon resonances. We focus on the inner properties of our modal sensor by immobilizing receptors directly on the silica surface, and reporting the sensitivity of bare tilted fiber Bragg gratings (TFBGs) used at near infrared wavelengths. We test different strategies to build our sensing surface against cytokeratins and show that the most reliable functionalization method is the electrostatic adsorption of antibodies on the fiber, allowing a limit of detection reaching 14 pM by following the guided cladding modes near the cut-off area. These results present the biodetection performance that TFBGs bring through their modal properties for different functionalizations and data processing strategies.

## 1. Introduction

Optical fiber biosensors are a growing center of interest thanks to their numerous advantages that have already been very widely described in the literature. Their low costs, high reliability, size, robustness, etc., are some of their many interests. They have also proven their biosensing skills, allowing the development of practical and minimally invasive tools for many types of bio-applications and for a large range of label-free molecular detections. In addition to that, they have reached very low limits of detection, earnestly competing with more conventional and commercially available biosensors [1,2].

However, a large number of these optical fiber biosensors exploit the surface plasmon resonance (SPR) phenomenon requiring the deposition of a thin metal film over their sensitive areas [3]. SPR consists of oscillations of electrons at the interface between positive and negative permittivity materials that are excited by incident light. These oscillations are sensitive to surface adsorption onto the metallic interface, often made of thin continuous films [4] or immobilized nanoparticles [5].

Over the last twenty years, SPR has been massively studied because the generation of surface plasmons increases the sensitivity of most of optical sensors [6]. To reach this sensitivity enhancement, perfect cleaning of the surface and precisely controlled gold deposition are needed to ensure a homogeneous deposit with a uniform thickness among the fiber surface. These gold depositions are often made by sputter-coaters or evaporators that need precise calibration. The gold layer thickness may also depend of the type of optical fiber used for experiments [7].

A hybrid phenomenon called “localized surface plasmon resonance” (LSPR) is also known to play a crucial role in sensing thanks to a higher light concentration occurring between metal nanoparticles and the analyzed sample, provoking an enhanced sensitivity due to the surface morphology [8]. In this case, the diameters and shapes of the nanoparticles are important parameters to manage in order to excite surface electrons localized at the metal interface [9].

Since the surface control is important, the inner properties of the optical fibers are also substantial to reach interesting biosensing features. Our group uses telecommunication-grade optical fibers where tilted fiber Bragg gratings (TFBGs) are photo-inscribed in the fiber core. These TFBGs can be used as physical sensors with growing interest in biomedical field [10,11,12], while their use as label-free SPR biosensors is possible when adding a continuous gold film of ~50 nm on the cladding surface [13]. Another important grating configuration for biosensing relies on long period fiber gratings (LPFGs) that excite cladding mode resonances in transmission mode [14,15]. Hence, techniques used for fiber surface functionalization such as those reported in this work are also relevant for this other sensing technology.

In this paper, we propose a refined approach consisting of an analysis of the intrinsic properties of our tilted gratings to demonstrate their characteristics and possibilities of biodetection with non-metallic surfaces. As a result, the absence of an intermediate conductive layer prevents SPR but still shows interesting properties for biosensing due to the high quality factor of the spectrum resonances involved in the detection scheme. We also propose three types of surface functionalizations, using three different coating processes to immobilize our receptors targeting cytokeratin proteins. A comparison between these three processes is also presented to select the most relevant method for detection using non plasmonic (bare) TFBGs in optical fibers.

Given the absence of any gold film, the biochemistry used for the immobilization of antibodies on the metal surface of SPR-assisted sensors can no longer be used and must be adapted for the pure silica glass of the optical fiber surface [16]. Fortunately, the binding of antibodies on glass has been the subject of an abundant literature and impressive progress have been realized for oriented or site-directed immobilizations [17]. However, biosensors need reliable binding of receptors, with a limited number of steps to achieve fast and repeatable process. We also notice that the advantages of biochemical strategies presented in the literature often differ, according to their final applications and pursued goals. This makes the transposition of functionalization protocols from one condition to another more complex, which explains why it is important to test the best molecular building blocks for our devices and to carefully consider the impact of the various conclusions reached in the many studies already published in that field. In the absence of the gold layer, several methods have been considered and adapted for use on optical fiber gratings. Three of them have been selected here as representative of three categories of molecular bonding.

The first immobilization process chosen is the covalent bonding of antibodies [18]. Covalent bonding is often presented as a stable graft, but antibodies can show reduced activity by forming linkage on active sites. It also needs a more complex chemistry [19,20].

The second is physisorption (physical adsorption) of the antibodies on the silica surface. These interactions are frequently exploited in biochemical assays (ELISA, antibody arrays, immune-sensors) because of their simplicity. Even if antibodies are randomly oriented and can lose their antigen binding abilities by denaturation, the passive adsorption often shows high antibody binding capacity. It is one of the main reasons why this functionalization is used for commercial assays [21]. The receptors can be conveniently adsorbed via intermolecular forces such as van der Waals, hydrophobic, electrostatic, hydrogen bonds, and their combinations [22].

The third functionalization is based on protein A/antibody affinity. Protein A is a well-known protein that targets the Fc region of antibodies. It helps to properly orient them for an optimum antigen binding. Immunoassays used with this configuration often show higher sensitivities, which is the main advantage of this method [23]. Unfortunately, this immobilization requires several biochemical steps that lengthen the sensor preparation and these affinity bonds are sometimes presented as less stable than simple covalent bonds [24].

In this work, we focus on the detection of cytokeratin protein because of its interest in lung cancer diagnosis. However, the functionalization remains open to any type of target, since the receptor immobilization is extremely flexible.

## 2. Materials and Methods

### 2.1. Materials

Phosphate Buffer Saline (PBS), (3-aminopropyl)trimethoxysilane 97% (APTMS) and sodium borohydride (NaBH_4_) were purchased from Sigma-Aldrich (Darmstadt, Germany). Anti-cytokeratin 17 antibodies (anti-CK17 ab), cytokeratin 7 (CK7) and cytokeratin 17 (CK17) full human proteins came from Abcam (Cambridge, UK). Bovine Serum Albumine (BSA) was purchased from Acros Organics (Geel, Belgium). All buffers were prepared freshly in deionized water and protein solutions were prepared in low-bind tubes in PBS at pH 7.4, and preserved at 4 °C until experiments.

### 2.2. Tilted Fiber Bragg Gratings Manufacturing

Telecommunication-grade optical fibers (SMF-28, 125 μm diameter, uncoated monomode silica optical fiber, Corning, New York, NY, USA) were used to connect each sensor. TFBGs were manufactured using a NORIA Laser (NorthLab Photonics, Nacka, Sweden) in photosensitive optical fiber (PS-1250, Fibercore, Southampton, UK). The grating manufacturing was performed using the deep ultraviolet pulsed excimer laser (Coherent Existar, XS 500 Hz-ArF at 193 nm) using the phase-mask technique to achieve 7° tilted gratings (uniform phase-mask with 5 mJ laser energy and 20.000 bursts at 50 Hz).

### 2.3. Optical Interrogation

All the experimental data (optical spectra) reported using bare-TFBGs were obtained in transmission, using a FiberSensing (Atlanta, GA, USA) 4CH interrogator. The optical fibers were immobilized on a fixed horizontal support and immersed successively in different liquid samples (from blank buffer to highest protein concentration).

### 2.4. Biosensor Surface Functionalization

Three different functionalization processes were used to immobilize our anti-CK17 antibodies on the silica surface. The common starting point of these processes is the thorough cleaning of the glass surface by immersing the fiber into a piranha solution made of H_2_SO_4_/H_2_O_2_ (4:1). Then, a silanization process is carried out to form a silane film on the surface, using immersion in (3-aminopropyl)trimethoxysilane (APTMS) 1% in methanol for 20 min at room temperature.

**Figure 1:**
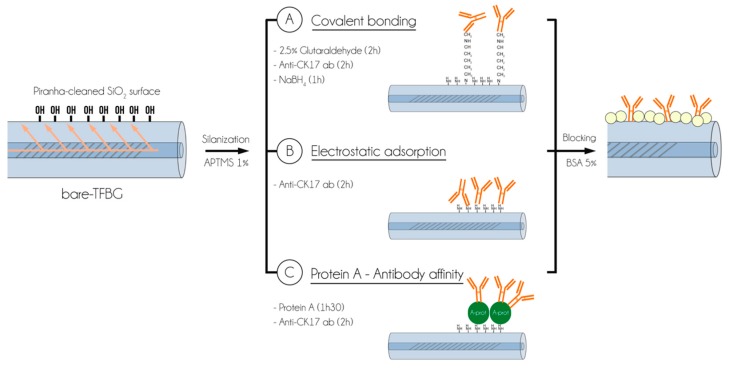
Illustration of the main steps of the three functionalization processes used for the immobilization of antibodies onto silica optical fibre gratings. The first method considered is a covalent binding of anti-CK17 antibodies (**A**) while electrostatic adsorption (**B**) and the use of Protein A intermediate are also represented (**C**).

For the first method of direct covalent binding of antibodies (Figure 1A), silanized TFBGs were first immersed in a 2.5% glutaraldehyde solution for 2 h at room temperature. Then, they were rinsed and immersed in anti-CK17 antibodies solution at 20 µg/mL in PBS during 2 h. After rinsing, they were finally immersed into 7.9 × 10^−3^% NaBH_4_ solution. This protocol was adapted from [25].

For the electrostatic immobilization, silanized optical fibers were directly immersed into anti-CK17 (20 µg/mL in PBS) solutions during 2 h at room temperature (Figure 1B). After that, TFBGs were gently rinsed using PBS and dried at RT. Our protocol was adapted from [26] and [27].

Finally, the third process consists of using an intermediate protein (Protein A) at 100 µg/mL in PBS, directly immobilized on the silica surface (Figure 1C). The protocol was adapted from [22].

All these functionalization processes were then finalized using a 5% BSA in PBS as blocking step at RT during 1 h30.

### 2.5. Data Analysis

The interrogation of TFBGs was performed through a LabView (National Instruments, Austin, TX, USA) 7.0 script, with automatic data acquisition. Then, the biosensing responses were analyzed using OriginLab 9.0 software (OriginLab, Northampton, MA, USA) and MATLAB (MathWorks, Natick, MA, USA). All the biosensing data presented were recorded after 5 min of exposure to target solutions, to ensure sufficient target coupling to the surface.

## 3. Results and Discussion

### 3.1. Stability in Deionized Water and PBS

**Figure 2:**
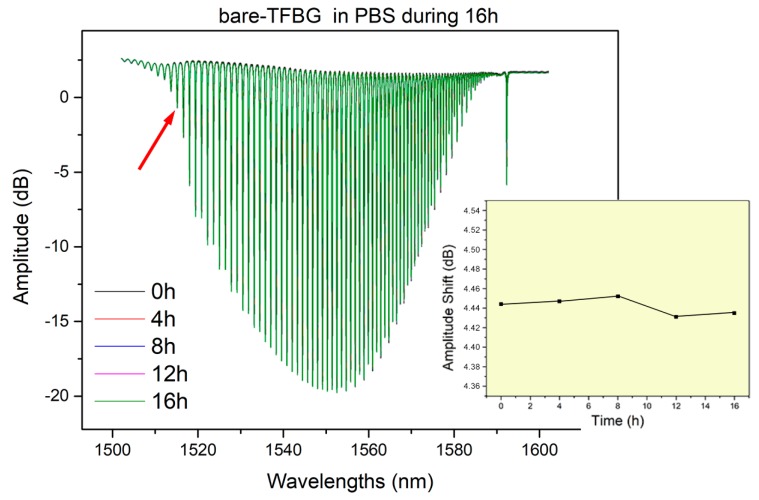
Graph showing bare tilted fiber Bragg gratings spectra after 4, 8, 12 and 16 h in PBS compared to initial measurement in same solution. The bio-responsive modes, which are tracked for biosensing and located near the cut-off wavelength are stable over time.

Bare-TFBGs, functionalized with anti-CK17 antibodies were immersed in solution for 16 h to test the stability of the signal for each biochemical strategy. Results show no significant variations of the biosensors responses, as illustrated in Figure 2.

### 3.2. Cytokeratin-17 Detection Experiments

The biosensitivity of our functionalized silica sensors was studied through laboratory tests using CK17 proteins in solution at increasing concentrations, from 10^−12^ g/mL up to 10^−6^ g/mL. The reference signal was recorded in PBS, which is also the buffer used for each CK17 dilution.

**Figure 3:**
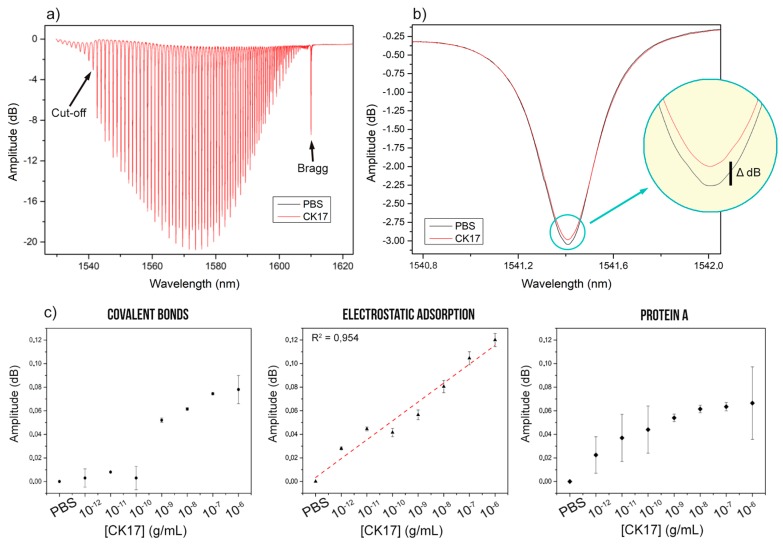
Near-IR spectra of one bare-TFBG functionalized with anti-CK17 antibodies, immersed into PBS and 10^−6^ CK17 solution, successively. (**a**) The first cut-off mode shows the most intense shift after immersion into CK17 solution. (**b**) After that, each functionalization process was tested for biosensing in growing CK17 concentration from 10^−12^ to 10^−6^ g/mL. Graphs show experimental results obtained in triplicates for each condition, using different sensors (mean ± standard deviation) with a linear fitting (red dotted line) for condition B (**c**).

A first observation is that for all our experiments, the most sensitive mode coupling resonance of the monitored near-IR spectral comb is located at the cut-off value. Indeed, when the external refractive index reaches the value of the effective refractive index of a given cladding mode, this mode is no longer guided and the resonance becomes a leaky one (the wavelength boundary between guide and leaky modes is the «cut-off wavelength») [28,29]. Because of the proximity of the effective index of the guided mode to the surrounding material refractive index, the evanescent field of this mode has the deepest penetration outside the fiber surface and makes the corresponding resonance the most sensitive to protein interactions with the fiber surface, while the guided modes further away from the cut-off show lower and eventually no sensitivity with increasing distance from the cut-off (Figure 3a,b).

The amplitude shifts of the cut-off resonance were then monitored after a compensation of any core mode resonance (the “Bragg” resonance of the grating) variation (in amplitude or wavelength), based on the fact that this resonance is immune to events on the fiber surface while its sensitivity to power level and temperature changes is similar to that of the “biosensing” resonance [30]. The difference in amplitude between maxima and minima of the cut-off resonance for each condition was also performed to avoid any baseline variations and to calculate the biosensitivity.

As expected, results show that the absence of surface plasmon excitation significantly reduces the sensing responses, which implies that a rigorous control of the fiber with constant environmental parameters, such as temperature and humidity is required to avoid signal pertubations. Optical fiber gratings also must be fixed on a support to prevent any movements during experiments.

Results indicate that the three different functionalizations can be effective for biosensing, but present different features (Figure 3c). For instance, covalent bonding of antibodies show a detection limit of ~10^−9^ g/mL with a high amplitude shift between 10^−10^ and 10^−9^ g/mL, while the electrostatic adsorption shows a more interesting LOD with constant shifts between each tested concentration. Finally, the use of Protein A intermediates shows concentration-dependent signal responses but seems to be less stable, leading to higher measurements errors and thus lowering its reproducibility. The latter can be explained by the complexity of the surface assembly of molecules and the increased distance between the silica surface and the location where the interaction of the target with the immobilized antibodies occurs, which is the source of the decrease in sensitivity in that case.

We prefer not to perform a comparative analysis of the performances between bare and gold-coated TFBGs. While plasmonic enhancements improve both the sensitivity and the LOD [13], bare gratings remain more suited for applications where a downscaling of the LOD is not required or whenever stability issues may prevent the use of plasmonic probes.

### 3.3. CK17 Specificity

**Figure 4:**
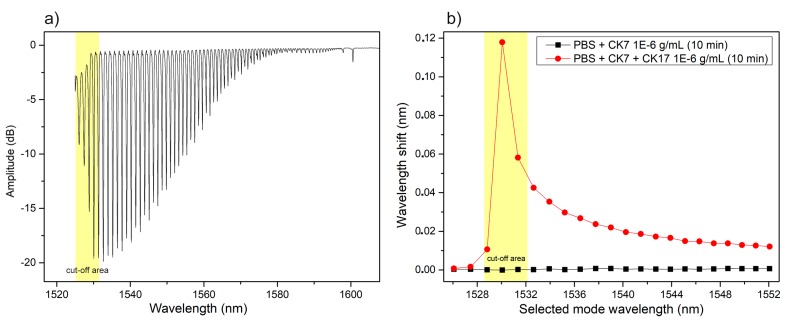
Cut-off area of Bare-TFBG spectrum. (**a**) The wavelength shifts do clearly indicate an increase in response towards the cut-off mode (near 1529 nm in this case) and also a total lack of response of the device exposed to a large concentration of the wrong target protein (CK7) referred as our negative control (**b**).

In this section, two other features of using bare-TFBG interrogation for biological detection are introduced. First, instead of using amplitude shifts of the resonance of the first cut-off mode, the resonance wavelength changes of all resonances in the vicinity of the cut-off, identified on the spectrum shown in Figure 4a (measured in PBS), are monitored. The rationale for that method is that the wavelengths of resonances approaching cut-off become increasingly sensitive to surrounding refractive index changes [31]. Second, the specificity of the detection of target proteins (CK17) relative to a closely related but different protein (CK7) is demonstrated using the wavelength shift method. Figure 4b shows how the wavelengths of the resonances identified in Figure 4a change from the reference spectrum in PBS after immersions for 10 min in a CK7 solution at 10^−6^ g/mL and then in a solution of 10^−6^ g/mL of CK17. The stability of the signal exposed to CK7 demonstrates the specificity of the sensor, a functional achievement obtained thanks to the surface-blocking step.

## 4. Conclusions

In this paper, we show our experimental results for the biodetection of cytokeratin-17 proteins using non-plasmon-assisted bare glass TFBGs. We demonstrate the feasibility of using non-metallic-coated surfaces for biodetection using the inherent increased sensitivity of the cladding modes located near the cut-off of the spectral comb, both in amplitude and wavelength. Furthermore, the functionalization process method chosen has a huge impact on the sensitivity of the sensor, with the electrostatic adsorption of antibodies seeming to be the fastest and the most effective grafting strategy. Covalent bonding shows larger modal shifts between 10^−10^ and 10^−9^ g/mL of CK17 in comparison to the progressive response for adsorbed antibodies. The use of intermediate linkers such as Protein A also shows concentration-dependent responses but with slightly lower modal shifts and higher measurements errors. This can be explained by the increasing distance between the target and the fiber surface due to the presence of the intermediate molecules, and the complexity of the functionalization process itself. The two other strategies show interesting features depending on the end-use application of the device. These findings could open the way to new biodetection strategies using optical fibers without the need to prepare high quality nanoscale metal coatings for SPR.
